# Influences of reconstruction and attenuation correction in brain SPECT images obtained by the hybrid SPECT/CT device: evaluation with a 3-dimensional brain phantom

**Published:** 2014

**Authors:** Mana Akamatsu, Yasuo Yamashita, Go Akamatsu, Yuji Tsutsui, Nobuyoshi Ohya, Yasuhiko Nakamura, Masayuki Sasaki

**Affiliations:** 1 Department of Medical Technology, Kyushu University Hospital, Japan; 2 Department of Health Sciences, Graduate School of Medical Sciences, Kyushu University, Japan; 3 Institute of Biomedical Research and Innovation, Japan

**Keywords:** Brain perfusion, Chang method, CTAC, Digital phantom, SPECT/CT

## Abstract

**Objective(s):**

The aim of this study was to evaluate the influences of reconstruction and attenuation correction on the differences in the radioactivity distributions in ^123^I brain SPECT obtained by the hybrid SPECT/CT device.

**Methods:**

We used the 3-dimensional (3D) brain phantom, which imitates the precise structure of gray matter, white matter and bone regions. It was filled with ^123^I solution (20.1 kBq/mL) in the gray matter region and with K_2_HPO_4_ in the bone region. The SPECT/CT data were acquired by the hybrid SPECT/CT device. SPECT images were reconstructed by using filtered back projection with uniform attenuation correction (FBP-uAC), 3D ordered-subsets expectation-maximization with uniform AC (3D-OSEM-uAC) and 3D OSEM with CT-based non-uniform AC (3D-OSEM-CTAC). We evaluated the differences in the radioactivity distributions among these reconstruction methods using a 3D digital phantom, which was developed from CT images of the 3D brain phantom, as a reference. The normalized mean square error (NMSE) and regional radioactivity were calculated to evaluate the similarity of SPECT images to the 3D digital phantom.

**Results:**

The NMSE values were 0.0811 in FBP-uAC, 0.0914 in 3D-OSEM-uAC and 0.0766 in 3D-OSEM-CTAC. The regional radioactivity of FBP-uAC was 11.5% lower in the middle cerebral artery territory, and that of 3D-OSEM-uAC was 5.8% higher in the anterior cerebral artery territory, compared with the digital phantom. On the other hand, that of 3D-OSEM-CTAC was 1.8% lower in all brain areas.

**Conclusion:**

By using the hybrid SPECT/CT device, the brain SPECT reconstructed by 3D-OSEM with CT attenuation correction can provide an accurate assessment of the distribution of brain radioactivity.

## Introduction

N-isopropyl-p-[^123^I] Iodoamphetamine (IMP) SPECT has been used to evaluate cerebrovascular disease, dementia and other brain disease (1–3). In brain perfusion SPECT, the attenuation and scatter of γ-rays emitted from inside the body are obstacles to an accurate assessment. There are several attenuation correction methods that have been developed. The Sorenson method is one of pre-reconstruction correction methods, where correction factors are applied to projection data before reconstruction, based on the geometric mean of the two opposing ray sums (4). The Chang method is one of post-correction methods, which allows for post-reconstruction correction with factors calculated from the pre-determined attenuation distribution (5). This method has been widely used to correct attenuation, because it is the easiest to apply among the many methods that have been developed. These methods assume that the brain has a single constant attenuation coefficient; however, the head consists of brain parenchyma, skull bone, nasal cavities and several other structures. Furthermore, during SPECT acquisition, the patient’s head is positioned on a head holder that may also affect photon attenuation (6).

Non-uniform attenuation correction methods have been developed to provide more precise attenuation correction (7, 8). A transmission computed tomography (TCT) method uses an external radiation source, which measures an attenuation map specific for each specific patient. Technical limitations associated with external sources have led to the development of CT based non-uniform attenuation correction (CTAC). CT images are initially obtained from independent machines and are matched to the SPECT image by software fusion (8).

Recently, hybrid SPECT/CT devices have been employed. SPECT/CT enables accurate attenuation correction, and can fuse perfusion and morphological images (9, 10). In a previous study reported by Ishii et al., the Chang and CTAC methods were compared in a clinical examination (6). However, it was a comparative study that the true radioactivity distributions were unknown. On the other hand, several studies have evaluated SPECT images using a digital phantom as a reference image (11, 12).

The purpose of this study was to evaluate the influences of reconstruction and attenuation correction on the radioactivity distributions in ^123^I brain SPECT obtained by the hybrid SPECT/CT device using a 3-dimensional (3D) brain phantom. The 3D digital phantom developed from CT image of the phantom was used as a reference.

## Methods

### The 3-dimensional Brain Phantom

**Figure 1:**
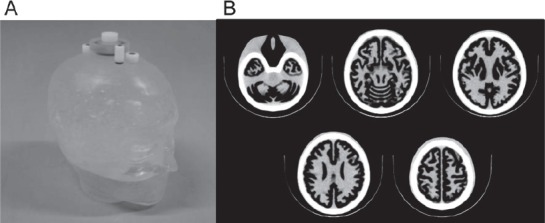
The 3D brain phantom: the appearance (A) and CT image (B). The phantom includes regions imitating the grey matter, white matter, cerebrospinal fluid space, skull and scalp. The grey matter can be filled with the radioactive solution

The 3D brain phantom (Molecular Imaging Labo Inc., Osaka, Japan) was developed by the National Cerebral and Cardiovascular Center Research Institute in Japan (Figure 1) (11). The phantom was constructed with a laser-modeling technique, and consists of a precise structure imitating the gray matter, white matter, cerebrospinal fluid space, skull and scalp based on MR images. The gray matter region was filled with ^123^I solution (20.1 kBq/mL). The white matter region, cerebrospinal fluid space and scalp were made of a photo-curable polymer. The bone region was filled with K_2_HPO_4_ (310.3 mL) (13).

### Imaging Protocol

#### Data Acquisition

The SPECT/CT data were acquired by a hybrid SPECT/CT device (Symbia T6, Siemens), a combination of a two-headed gamma camera and a 6-slice multi-detector row CT. The matrix size was 128×128, with a pixel size of 3.9 mm. The data acquisition was performed by a continuous clockwise and counter clockwise rotation over 180° by five rotations of five minutes each. The main energy window was 20%, with an ^123^I photopeak of 159 keV. The sub-windows were 7% above and below the main window. The triple energy window (TEW) method was used for scatter correction in all reconstruction methods.

The CT scan protocol was performed using the following parameters: 130 kV, 55 mAs, 0.6 s tube rotation, 3 mm slice collimation and a 300 mm field-of-view.

#### Image Reconstruction

**Figure 2:**
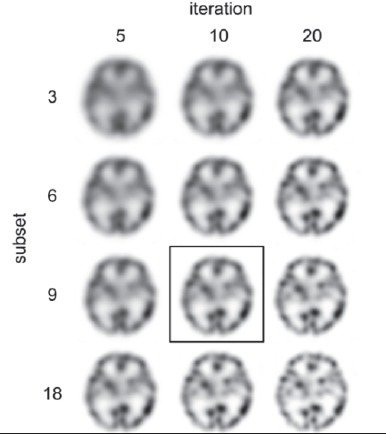
The SPECT images by 3D-OSEM-CTAC with various reconstruction parameters. We chose the iteration of 10 and subset of 9 based on the visual assessment of the balance between spatial resolution and noise for the SPECT images

The SPECT images were reconstructed by using FBP and 3D-OSEM. A ramp filter was applied for FBP reconstruction. The 3D-OSEM incorporates collimator depth-dependent three-dimensional resolution recovery compensation (12). The reconstruction parameter for OSEM was 10 iterations and 9 subsets (Figure 2). The Chang attenuation correction assumed a uniform attenuation coefficient of 0.12 cm^-1^. The CT-based non-uniform attenuation correction used an attenuation map which was obtained from the CT images. We supplementarily obtained the mean μ values of the head holder, water and bone from the attenuation map. As a smoothing filter, a Butterworth filter (cutoff: 0.36 cycles/2 pixels, order 8) was used in FBP and a Gaussian filter (a full width at a half maximum of 7.8 mm) in 3D-OSEM.

The attenuation coefficient of Chang method was determined by preliminary pool phantom evaluation. The diameter of the pool phantom was 16 cm. The attenuation coefficient was varied from 0.09 to 0.17. The uniformity of the pool phantom image was evaluated by visual assessment for profile flatness (5 grades: -2, -1, 0, +1, +2) by 5 radiological technologists. We also placed a 16-cm-diameter circular ROI on the phantom image and calculated the coefficient of variation (CV). The best flatness of the profile curve with 0 was obtained at attenuation coefficient of 0.12 cm^-1^. The smallest CV of 6.8% was obtained for the image with attenuation coefficient of 0.12 cm^-1^. Thus, the attenuation coefficient of 0.12 cm^-1^ was used for this study.

The reconstruction parameters for OSEM were determined by visual assessment evaluating the image quality of the SPECT images by 3 nuclear medicine physician and 5 radiological technologists from the point of view of spatial resolution and uniformity.

In this study, we evaluated the SPECT images reconstructed by using FBP with uniform attenuation correction (FBP-uAC), 3D-OSEM with uniform AC (3D-OSEM-uAC) and CT-based non-uniform AC (3D-OSEM-CTAC).

### Development of the 3D Digital Brain Phantom

**Figure 3:**
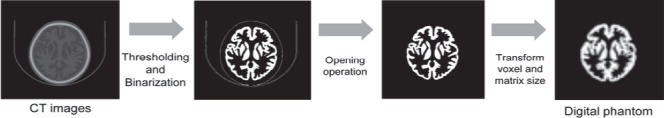
The flow chart of the development of the 3D digital brain phantom from CT images of the 3D brain phantom. All processes were performed using the ImageJ software program

The 3D digital brain phantom was developed from CT images of the 3-D brain phantom by using the ImageJ software program (National Institutes of Health, Bethesda, Maryland, USA). The gray matter region with water density in the CT images was extracted by image processing, thresholding techniques and an opening operation. The image matrix of the binarized gray matter region was then changed to the same matrix size as the SPECT images. Finally, a post-processing smoothing filter equal to the SPECT images was applied to obtain the 3D digital brain phantom (Figure 3).

### Data Analysis

#### Normalized mean squared error (NMSE)

The NMSE was calculated in order to evaluate the deviation between the SPECT images and the 3D digital phantom on the basal ganglia level (13, 14). We calculated the NMSE of the axial images as follows:


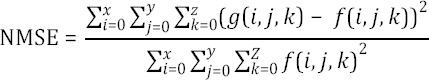


where *f* (i, j, k) and *g* (i, j, k) represent the pixel values of the reference and target images.

#### Regional radioactivity

**Figure 4:**
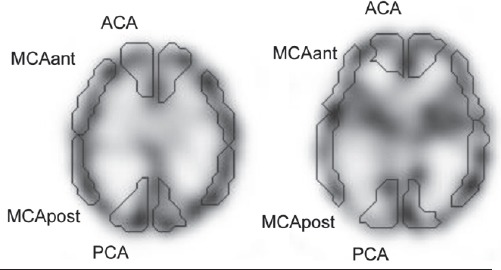
The regions of interest identified by NEURO FLEXER program. ACA: anterior cerebral artery territory, MCA ant: anterior-middle cerebral artery territory, MCA post: posterior-middle cerebral artery territory, PCA: posterior cerebral artery territory

We also examined the differences in the regional radioactivities among the SPECT images using a digital phantom as a reference. For the analysis, the template ROIs was automatically set by using the NEURO FLEXER program (Nihon Medi-physics Co., Ltd., Tokyo, Japan) by referring to the Talairach atlas (15). The acquired SPECT images were anatomically standardized to obtain information of the voxel transformation. Based on this information, the ROIs template was modified to the SPECT images by inverse transformation. The ROI counts were then normalized to relative radioactivity as a reference of the maximum count. The ROIs of the anterior cerebral artery (ACA), anterior-middle cerebral artery (MCAant), posterior-middle cerebral artery (MCApost) and posterior cerebral artery (PCA) territories were used for the analysis (Figure 4).

#### Visual assessment

For visual assessment, the SPECT images were evaluated according to the consistency with the digital phantom on a 3-step scale (1, poor; 2, fair; 3, good) by 1 board-certified nuclear physician and 3 radiological technologists.

## Results

**Figure 5:**
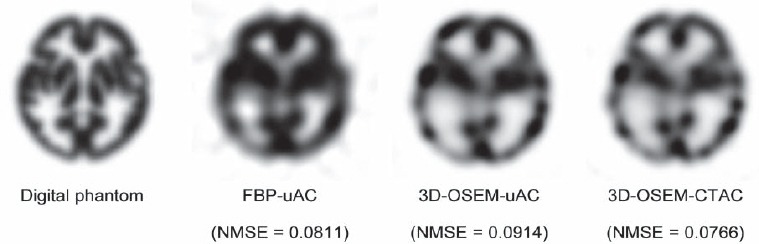
The SPECT images and NMSE values. The 3D-OSEM-CTAC image was the closest to the digital phantom

The NMSE value were 0.0820 in FBP-uAC, 0.0895 in 3D-OSEM-uAC and 0.0734 in 3D-OSEM-CTAC (Figure 5). The 3D-OSEM-CTAC image was considered to be the most similar to the reference image of the digital phantom.

**Figure 6:**
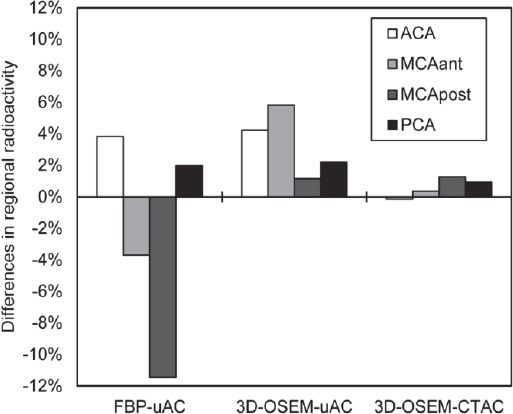
The differences in the regional radioactivity of FBP-uAC, 3D-OSEM-uAC and 3D-OSEM-CTAC in comparison to that of the digital phantom. The differences between the digital phantom and the 3D-OSEM-CTAC values were 1.8% or less in all brain regions

In comparison with the digital phantom, the regional radioactivity in FBP-uAC was higher in the ACA and PCA, while it was lower in the MCAant and MCApost (Figure 6). The difference was the largest in the MCApost (-11.5%). The

In comparison with the digital phantom, the regional radioactivity in FBP-uAC was higher in the ACA and PCA, while it was lower in the MCAant and MCApost (Figure 6). The difference was the largest in the MCApost (-11.5%). The regional radioactivity in all cerebral cortices in 3D-OSEM-uAC was higher than that of the digital phantom (range from 1.2 to 5.8%). In the 3D-OSEM-CTAC, the difference in the radioactivity was 1.8% or less in all regions.

The mean visual scores were 1.5 for all three SPECT images. The mean μ value of the head holder, water, and bone were 0.12, 0.12 and 0.21 cm^-1^ which were obtained from the attenuation map.

## Discussion

In this study, we evaluated the influences of reconstruction and attenuation correction on the radioactivity distributions in ^123^I brain SPECT obtained by the hybrid SPECT/CT device with reference to the 3-D digital phantom. Both the NMSE and regional radioactivity suggested that the SPECT image of 3D-OSEM-CTAC was the closest to the reference.

The NMSE of 3D-OSEM-CTAC was the smallest among 3 images. On the other hand, the NMSE values of FBP-uAC were equivalent to those of 3D-OSEM-uAC. Furthermore, the regional activity in the 3D-OSEM-CTAC images was the closest to that of the digital phantom. The regional activities of the ACA area in FBP-uAC and 3D-OSEM-uAC were similarly higher than those of the digital phantom. Ishii et al. also reported that counts in the frontal area of the Chang’s-AC images were significantly higher than those in the CTAC images (6). In a simulation study reported by Arlig et al., the frontal lobe activity in the Chang’s-AC images was overestimated compared to that in the CTAC images (14). Our results also suggested that the frontal counts of the Chang’s-AC images were higher than those of the CTAC images. Decreased frontal counts in the CTAC image may relate to consider the attenuation of the head holder (6).

When we compared the regional activities between FBP-uAC and 3D-OSEM-uAC, the relative activities in the MCA area were considerably different. In FBP-uAC images, that of the MCApost was underestimated by 11.5%. On the other hand, in 3D-OSEM-uAC images, that of the MCAant was overestimated. Several studies have reported that OSEM provided better spatial resolution compared to FBP (12, 16–18). The MCA area has complicated regions, which can lead to underestimation of the activity in the small regions by the partial volume effect. On the other hand, the image reconstructed with the FBP has lower resolution. Thus, the MCA area of the FBP image could be underestimated by the partial volume effect. Furthermore, 3D-OSEM includes resolution recovery compensation. OSEM with the resolution recovery method has previously been shown to improve the spatial resolution and contrast (12, 18).

Our results showed that the 3D-OSEM-CTAC image was the most similar to the 3-D digital brain phantom among the three SPECT images. Recently, the digital phantom has been used as a reference image for SPECT examinations (12, 19). Our proposed digital phantom can be easily developed and applied for such examinations. The CTAC method can estimate the non-uniform attenuation of both the head and head holder at the same time. Therefore, the CTAC method is considered to provide the most accurate attenuation correction for measuring the true regional radioactivity.

Although comparative studies of Chang’s and CT attenuation correction methods in brain perfusion SPECT have been performed in several previous studies, the CT used in their studies were separate devices (7, 8, 14). Using separate CT involves the risk of a mismatch between the CT and SPECT images (20). In contrast, the spatial mismatch between the CT and SPECT images is negligible when using a hybrid SPECT/CT device. Thus, using hybrid SPECT/CT device for CT attenuation correction is considered to provide more accurate brain perfusion distributions.

This study had limitations that should be kept in mind when interpreting the results. First, the white matter region in the 3-D brain phantom could not be filled with the radioactive solution. A further study is required to evaluate the clinical brain SPECT images and to confirm that the present phantom results can be translated to the clinical setting. Second, we used the reconstruction parameters determined by our phantom examination. The iteration and subset in OSEM are known to influence the regional radioactivity (12). When we evaluate various images, the standardization of the reconstruction parameters is considered to be important among the reconstruction methods. Third, the visual inspection is not corresponded to the results of NMSE value. It is not greatly improved the image quality visually. Moreover, it is considered to depend on the subjectivity of the reading physician.

## Conclusion

The 3D-OSEM with CT attenuation correction using hybrid SPECT/CT is considered to provide an accurate assessment of the brain radioactivity distribution. In addition, our proposed digital phantom could be used to accurately evaluate the SPECT/CT images.

## References

[1] Hayashida K, Nishimura T, Imakita S, Uehara T, Nakamura M, Tsuchiya T (1991). Change of accumulation and filling pattern in evolution of cerebral infarction with I-123 IMP brain SPECT. Neuroradiology.

[2] Sharp P, Gemmell H, Cherryman G, Besson J, Crawford J, Smith F (1986). Application of iodine-123-labeled isopropylamphetamine imaging to the study of dementia. J Nucl Med.

[3] Akiyama Y, Moritake K, Yamasaki T, Kimura Y, Kaneko A, Yamamoto Y (2000). The diagnostic value of ^123^I-IMP SPECT in non-Hodgkin's lymphoma of the central nervous system. J Nucl Med.

[4] Sorenson JA (1974). Methods for quantitative measurement of radioactivity in vivo by whole-body counting.

[5] >Chang LT (1978). A method for attenuation correction in radionuclide computed tomography. IEEE Trans Nucl Sci.

[6] Ishii K, Hanaoka K, Okada M, Kumano S, Komeya Y, Tsuchiya N (2012). Impact of CT attenuation correction by SPECT/CT in brain perfusion images. Ann Nucl Med.

[7] Stodilka RZ, Kemp BJ, Prato FS, Nicholson RL (1998). Importance of bone attenuation in brain SPECT quantification. J Nucl Med.

[8] Van Laere K, Koole M, Versijpt J, Dierckx R (2001). Non-uniform versus uniform attenuation correction in brain perfusion SPET of healthy volunteers. Eur J Nucl Med.

[9] Lang TF, Hasegawa BH, Liew SC, Brown JK, Blankespoor SC, Reilly SM (1992). Description of a prototype emission-transmission computed tomography imaging system. J Nucl Med.

[10] Patton JA, Turkington TG (2008). SPECT/CT physical principles and attenuation correction. J Nucl Med Technol.

[11] Iida H, Hori Y, Ishida K, Imabayashi E, Matsuda H, Takahashi M (2013). Three-dimensional brain phantom containing bone and grey matter structures with a realistic head contour. Ann Nucl Med.

[12] Onishi H, Motomura N, Fujino K, Natsume T, Haramoto Y (2013). Quantitative performance of advanced resolution recovery strategies on SPECT images: evaluation with use of digital phantom models. Radiol Phys Technol.

[13] de Dreuille O, Strijckmans V, Almeida P, Loc’h C, Bendriem B (1997). Bone equivalent liquid solution to assess accuracy of transmission measurements in SPECT and PET. IEEE Trans Nucl Sci.

[14] Arlig A, Gustafsson A, Jacobsson L, Ljungberg M, Wikkelsö C (2000). Attenuation correction in quantitative SPECT of cerebral blood flow: a Monte Carlo study. Phys Med Biol.

[15] Ogura T, Hida K, Masuzuka T, Saito H, Minoshima S, Nishikawa K (2009). An automated ROI setting method using NEUROSTAT on cerebral blood flow SPECT images. Ann Nucl Med.

[16] Brambilla M, Cannillo B, Dominietto M, Leva L, Secco C, Inglese E (2005). Characterization of ordered-subsets expectation maximization with 3D post-reconstruction Gauss filtering and comparison with filtered backprojection in ^99m^Tc SPECT. Ann Nucl Med.

[17] Yokoi T, Shinohara H, Onishi H (2002). Performance evaluation of OSEM reconstruction algorithm incorporating three-dimensional distance-dependent resolution recovery compensation for brain SPECT: A simulation study. Ann Nucl Med.

[18] Knoll P, Kotalova D, Köchle G, Kuzelka I, Minear G, Mirzaei S (2012). Comparison of advanced iterative reconstruction methods for SPECT/CT. Z Med Phys.

[19] Onishi H, Matsutake Y, Kawashima H, Matsutomo N, Amijima H (2011). Comparative study of anatomical normalization errors in SPM and 3D-SSP using digital brain phantom. Ann Nucl Med.

[20] Larsson A, Johansson L, Sundstrom T, Ahlström KR (2003). A method for attenuation and scatter correction of brain SPECT based on computed tomography images. Nucl Med Commun.

